# Three-Way Junction-Induced Isothermal Amplification with High Signal-to-Background Ratio for Detection of Pathogenic Bacteria

**DOI:** 10.3390/s21124132

**Published:** 2021-06-16

**Authors:** Jung Ho Kim, Seokjoon Kim, Sung Hyun Hwang, Tae Hwi Yoon, Jung Soo Park, Eun Sung Lee, Jisu Woo, Ki Soo Park

**Affiliations:** Department of Biological Engineering, College of Engineering, Konkuk University, Seoul 05029, Korea; jung9511@konkuk.ac.kr (J.H.K.); ghjghy@konkuk.ac.kr (S.K.); tjdgus0410@konkuk.ac.kr (S.H.H.); yth4784@konkuk.ac.kr (T.H.Y.); pjs219@konkuk.ac.kr (J.S.P.); afish94@konkuk.ac.kr (E.S.L.); wojam21@konkuk.ac.kr (J.W.)

**Keywords:** pathogen, detection, nucleic acids, three-way junction-induced isothermal amplification

## Abstract

The consumption of water and food contaminated by pathogens is a major cause of numerous diseases and deaths globally. To control pathogen contamination and reduce the risk of illness, a system is required that can quickly detect and monitor target pathogens. We developed a simple and reproducible strategy, termed three-way junction (3WJ)-induced transcription amplification, to detect target nucleic acids by rationally combining 3WJ-induced isothermal amplification with a light-up RNA aptamer. In principle, the presence of the target nucleic acid generates a large number of light-up RNA aptamers (Spinach aptamers) through strand displacement and transcription amplification for 2 h at 37 °C. The resulting Spinach RNA aptamers specifically bind to fluorogens such as 3,5-difluoro-4-hydroxybenzylidene imidazolinone and emit a highly enhanced fluorescence signal, which is clearly distinguished from the signal emitted in the absence of the target nucleic acid. With the proposed strategy, concentrations of target nucleic acids selected from the genome of *Salmonella* *enterica* serovar Typhi (*S.* Typhi) were quantitatively determined with high selectivity. In addition, the practical applicability of the method was demonstrated by performing spike-and-recovery experiments with *S*. Typhi in human serum.

## 1. Introduction

According to the World Health Organization (WHO), more than 2.2 million people die each year globally as a result of waterborne diseases, of which approximately 1.4 million are children, leading to a significant economic loss of about $12 billion [1,2,3]. The spread of pathogens and disease should be controlled to mitigate the incurred economic and social burden, resulting in high demand for a system that can accurately detect and regularly monitor pathogens [3,4]. The gold standards for detecting pathogens are bacterial culture and biochemical tests [5]. However, these methods have critical limitations, including the need for sophisticated and time-consuming experimental procedures, incompatibility of microorganisms with bacterial culture, and long turnaround time for results (2 or 3 days) [4,5,6,7]. As an alternative, researchers have focused on nucleic acid-based assays. One of the most popular and well-established techniques is polymerase chain reaction (PCR), which can detect even a single copy of target nucleic acids [4,5]. Currently, PCR is widely used for the detection of severe acute respiratory syndrome coronavirus 2 (SARS-CoV-2) [8,9]. However, despite its excellent sensitivity and selectivity, PCR relies on repeated thermal cycling to amplify the target nucleic acids and, therefore, is unsuitable for application in facility-limited environments or point-of-care settings [10,11]. 

Recently, several types of isothermal amplification methods that do not require thermal cycling have received increased attention [8,11,12,13,14,15,16,17,18,19,20,21,22,23,24,25]. In particular, the three-way junction (3WJ)-induced isothermal amplification (ThIsAmp) technique was developed for the sensitive and selective detection of long-stranded genomic DNA [26]. In principle, the presence of target DNA induces the formation of the 3WJ structure, which subsequently initiates the exponential amplification reaction (EXPAR) mediated by DNA polymerase and a nicking endonuclease [26]. The ThIsAmp process is performed at 55 °C and monitored through SYBR Green (SG) I staining, overcoming the limitations of conventional EXPAR [26]. 

Herein, we developed a new strategy, termed 3WJ-induced transcription amplification, with a high signal-to-background (S/B) ratio that advances ThIsAmp. Rather than using SG I, which is specific to double-stranded (ds) DNA without sequence selectivity, a light-up RNA aptamer was rationally adopted through a process of in vitro transcription reaction [27,28,29,30,31]. Because the light-up RNA aptamer specifically binds to its cognate fluorogen, substantially increasing its fluorescence signal [30,32,33,34,35], we hypothesized that this would increase the S/B ratio and reduce the generation of a false positive signal induced by ab initio synthesis [36,37,38]. In addition, the system was tailored to operate at 37 °C, which is more desirable than 55 °C for field applications. As proof of concept, we optimized the conditions for 3WJ-induced transcription amplification and demonstrated the utility of the method by detecting target nucleic acids of *Salmonella* *enterica* serovar Typhi (ST) [39,40], one of the main pathogens that causes food poisoning.

## 2. Material and Methods

### 2.1. Materials

All DNA oligonucleotides used in this study were purchased from Integrated DNA Technologies (Skokie, IL, USA). The oligonucleotide sequences are listed in Table 1. Klenow DNA polymerase (exo-) and T7 RNA polymerase were purchased from Enzynomics Inc. (Daejeon, Korea). The nicking endonuclease Nt.AlwI and Cutsmart^®^ buffer were purchased from New England Biolabs Inc. (Ipswich, MA, USA). The human serum and fluorogen 3,5-difluoro-4-hydroxybenzylidene imidazolinone (DFHBI) that binds to the Spinach aptamer were purchased from Sigma-Aldrich (St Louis, MO, USA). SYBR™ Green I (SG I), SYBR™ Green II (SG II), and SYBR Safe (SS) were purchased from Thermo Fisher Scientific (Waltham, MA, USA). All other chemicals were of analytical grade and used without further purification.

### 2.2. Detection Procedure for Target Nucleic Acids

The 3WJ-induced transcription amplification reaction was performed in two steps: DNA amplification followed by transcription amplification. For DNA amplification, solution A containing 4 μL of 250 nM 3WJ_template, 8 μL of 250 nM 3WJ_primer, 1 μL of 10 mM (each 2.5 mM) deoxyribonucleotide triphosphates (dNTPs), 10 μL of 10 mM (each 2.5 mM) nucleotide triphosphates (NTPs), and 4 μL of 10× Cutsmart^®^ buffer was incubated with the target DNA at different concentrations for 5 min at 37 °C. To this mixture, solution B containing 0.4 μL of 10 U/μL Klenow DNA polymerase (exo-) and 0.8 μL of 10 U/μL Nt.AlwI was added and incubated for 1 h at 37 °C. For transcription amplification, 1.2 μL of 50 U/μL T7 RNA polymerase was added to a total reaction volume of 40 μL and incubated for 1 h at 37 °C. Finally, 10 μL DFHBI (50 μM) was added to interact with the light-up RNA aptamer and generate a fluorescence signal, which was measured at an excitation wavelength of 452 nm and an emission wavelength of 506 nm using a SpectraMax iD5 microplate reader (Molecular Devices, Sunnyvale, CA, USA).

### 2.3. Polyacrylamide Gel Electrophoresis (PAGE)

The reaction products were resolved on 15% polyacrylamide gel using 1× Tris/borate/EDTA (TBE) as a running buffer at a constant voltage of 100 V for 150 min. After staining with GreenStar (Bioneer, Daejeon, Korea), the gel image was acquired using the FAS-Nano gel documentation system (Nippon Genetics, Binsfelder, Germany).

### 2.4. Melting Curve Analysis

The reaction products in the presence of 1× SG I were incubated for 1 h at 37 °C. The fluorescence signal was measured using the CFX Connect Real-Time PCR Detection System (Bio-Rad, Hercules, CA, USA) as the temperature was increased from 50 to 95 °C in increments of 0.5 °C. A first derivative plot (-d(RFU)/dT) was used to determine the melting temperature.

### 2.5. Detection Procedure for Target Nucleic Acids in Human Serum

To assess the practical application of the proposed strategy, varying concentrations of the target DNA were spiked in diluted human serum (1%) and subjected to the same procedure as described above.

## 3. Results and Discussion

### 3.1. Overall Detection Procedure

**Figure 1:**
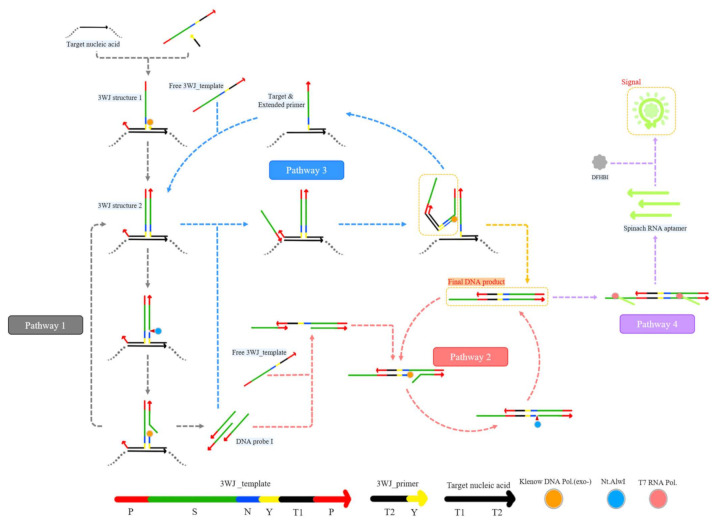
Schematic illustration of a 3WJ-induced transcription amplification for the detection of target nucleic acids. P, promoter sequence (red); S, light-up RNA aptamer sequence (green); N, recognition site for Nt.AlwI (blue); Y, sequence that mediates the formation of 3WJ (yellow); T1, target-specific region (black); T2, target-specific region (black).

Figure 1 illustrates the 3WJ-induced transcription amplification process. The template (3WJ_template) contains the light-up RNA aptamer sequence (S, green), the recognition site for Nt.AlwI (5’-NˇNNNNGATCC-3’, N, blue), a sequence that mediates the formation of 3WJ (Y, yellow), the target-specific region (T1, black), and a promoter sequence (P, red). In addition, the primer (3WJ_primer) contains a target-specific region (T2, black) and a sequence that mediates the formation of 3WJ (Y, yellow). The presence of the target nucleic acid (T1-T2, black) induces the formation of the 3WJ structure with 3WJ_template and 3WJ_primer, leading to the strand displacement amplification reactions by Klenow DNA polymerase (exo-) and Nt.AlwI. As a result, abundant DNA probe I is produced in pathway 1. Furthermore, DNA probe I binds to 3WJ_template in pathways 2 and 3, initiating multiple rounds of strand displacement amplification reactions. Importantly, strand displacement amplification (pathways 1–3) is combined with T7 RNA polymerase-mediated transcription amplification (pathway 4). Because the final DNA product contains two ds promoter regions, T7 RNA polymerase initiates transcription amplification. As a result, several light-up RNA aptamers (Spinach aptamers) are generated, leading to a high fluorescence signal after complexation with DFHBI (pathway 4). In contrast, in the absence of the target nucleic acids, the 3WJ structure is not formed, and thus 3WJ-induced transcription amplification does not occur, emitting a negligible fluorescence signal. The proposed strategy is performed at 37 °C, and the target nucleic acids can be identified within 2 h by measuring the fluorescence signal emitted by DFHBI.

### 3.2. Detection Feasibility of the Proposed Strategy

**Figure 2:**
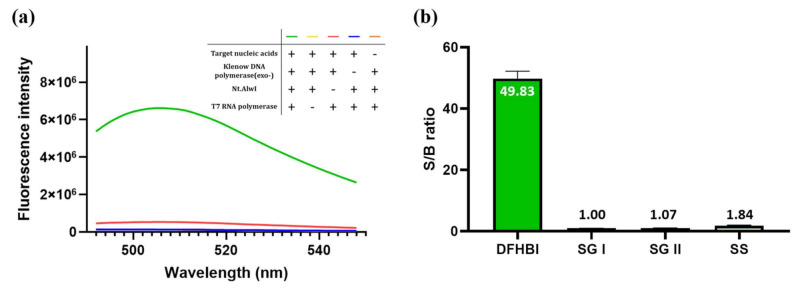
Detection feasibility of the proposed strategy. (**a**) Fluorescence emission spectra under different conditions. (**b**) The signal-to-background (S/B) ratio of the proposed strategy using different nucleic acid staining dyes such as DFHBI, SYBR Green I (SG I), SYBR Green II (SG II), and SYBR Safe (SS). All experiments were performed in triplicate.

To confirm the detection feasibility of the proposed strategy, we first measured the fluorescence signal of DFHBI under different conditions. As shown in Figure 2a, the presence of the target nucleic acids generated the highest fluorescence signal only when all enzymes (Klenow DNA polymerase (exo-), Nt.AlwI, and T7 RNA polymerase) required for 3WJ-induced transcription amplification were present (green, yellow, red, and blue curves). When Nt.AlwI was excluded (red curve), a slightly intense fluorescence signal was produced, which was attributed to the formation of the ds promoter region by Klenow DNA polymerase (exo-); however, this fluorescence signal was much lower than that produced in the presence of all enzymes. Most importantly, when the target nucleic acids were absent (orange curve), 3WJ-induced transcription amplification was not initiated, which was manifested by a negligible fluorescence signal.

Next, we compared our system with commonly used fluorescent dyes such as SG I, SG II, and SS. The signal-to-background (S/B) ratio, defined as (F-F_0_)/F_0_, where F_0_ and F are the fluorescence signals in the absence and presence of the target nucleic acids, respectively, was approximately 1–2 when SG I, SG II, or SS was used (Figure 2b). In contrast, our system produced a dramatically increased S/B ratio of 49.8. We hypothesized that common fluorescent dyes lacking sequence specificity produced high background signals by interacting with non-specific amplification products generated by ab initio synthesis, whereas the light-up RNA aptamers in our system discriminated non-specific amplification products, proving that the proposed system reduced the chance of a false positive signal.

### 3.3. Mechanism Investigation of the Proposed Strategy

**Figure 3:**
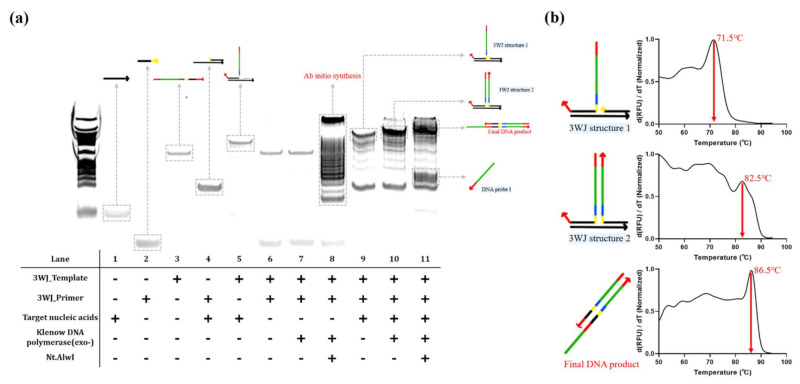
Confirmation of 3WJ-induced isothermal amplification. (**a**) Representative images of polyacrylamide gel electrophoresis of the reaction products obtained after strand displacement amplification. (**b**) Melting curve analysis for the reaction products obtained after strand displacement amplification. 3WJ structure 1: target DNA + 3WJ_template + 3WJ_primer (lane 9 in (**a**)); 3WJ structure 2: target DNA + 3WJ_template + 3WJ_primer + Klenow DNA polymerase (exo-) (lane 10 in (**a**)); final DNA product: target DNA + 3WJ_template + 3WJ_primer + Klenow DNA polymerase (exo-) + Nt.AlwI (lane 11 in (**a**)).

We conducted PAGE analysis to investigate the mechanism of the proposed strategy. As shown in Figure 3a, the target nucleic acids, 3WJ_template, and 3WJ_primer formed 3WJ structure 1 (lanes 1, 2, 3, and 9). In addition, 3WJ structure 2 was formed in which 3WJ_primer was extended from 3WJ structure 1 by Klenow DNA polymerase (exo-), and the final DNA product was obtained by Klenow DNA polymerase (exo-) and Nt.AlwI (lanes 10 and 11). Importantly, in the absence of the target nucleic acids, 3WJ_primer and 3WJ_template did not form 3WJ structure 1, preventing the extension reaction by Klenow DNA polymerase (exo-) (lanes 6 and 7). However, the presence of both Klenow DNA polymerase (exo-) and Nt.AlwI, even in the absence of the target nucleic acids, produced non-specific amplification products that arose from ab initio synthesis (lane 8). These results concurred with the results shown in Figure 2b. We also performed melting curve analysis to further confirm these results. As shown in Figure 3b, the sample corresponding to lane 9 in Figure 3a generated a single melting peak of 3WJ structure 1 (71.5 °C). In addition, the samples corresponding to lanes 10 and 11 in Figure 3a generated the melting peaks of 3WJ structure 2 (82.5 °C) and final DNA product (86.5 °C), respectively. Taken together, these results confirmed that 3WJ structures were effectively formed in the presence of the target nucleic acids, and the isothermal amplification occurred as proposed in Figure 1.

### 3.4. Optimization of Reaction Conditions

**Figure 4:**
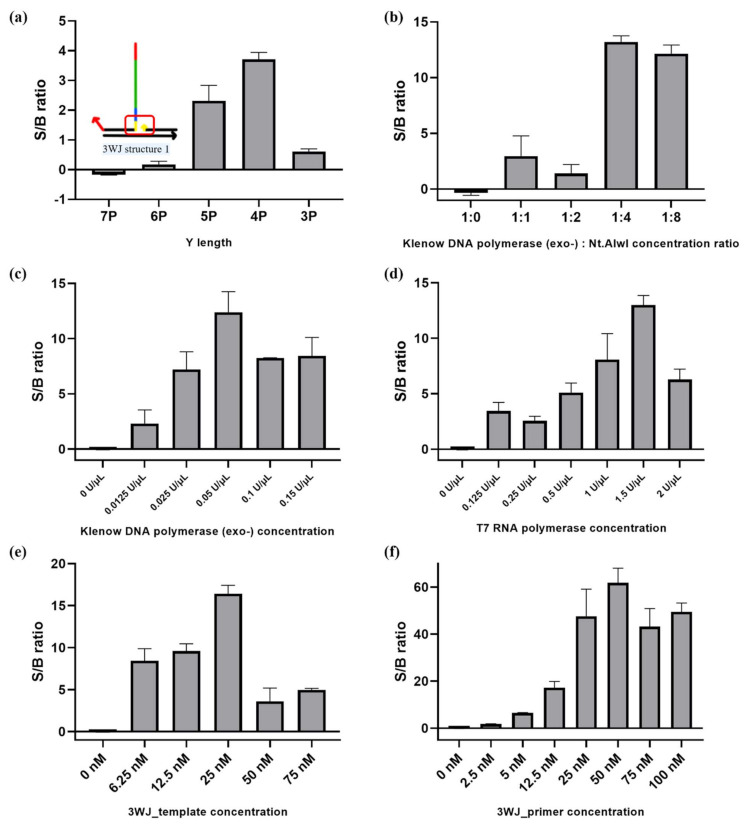
Optimization of 3WJ-induced transcription amplification. (**a**) The effect of complementary sequence (Y) length between 3WJ_primer and 3WJ_template. (**b**) Optimization of the Klenow DNA polymerase (exo-) and Nt.AlwI ratio. (**c**) Optimization of Klenow DNA polymerase (exo-) concentration. The Klenow DNA polymerase (exo-) and Nt.AlwI ratio is 1:4. (**d**) Optimization of T7 RNA polymerase concentration. (**e**) Optimization of 3WJ_template concentration. (**f**) Optimization of 3WJ_primer concentration. The concentration of the target nucleic acids is 10 nM. All experiments were performed in triplicate.

First, we optimized the complementary sequence length between 3WJ_primer and 3WJ_template. The results in Figure 4a demonstrate that the S/B ratio decreased as the complementary sequence length was increased up to 7 base pairs (bp), which was attributed to the ability of 3WJ_primer and 3WJ_template to hybridize each other even in the absence of the target nucleic acids, thus increasing the background signal. On the contrary, when the sequence length was short (3 bp), the 3WJ structure was not effectively formed even in the presence of the target nucleic acids, leading to a low S/B ratio. Based on these optimization results, the complementary sequence length between 3WJ_primer and 3WJ_template was selected as 4 bp. 

Next, reaction conditions such as enzyme and DNA probe concentrations were optimized to maximize the detection efficiency of the proposed strategy by comparing fluorescence signals in the absence and presence of the target nucleic acids. The optimal reaction conditions were found to be as follows: Klenow DNA polymerase (exo-), 0.05 U/μL; Nt.AlwI, 0.2 U/μL; T7 RNA polymerase, 1.5 U/μL; 3WJ_template, 25 nM; and 3WJ_primer, 50 nM. These optimized reaction conditions were subsequently used for further experiments. 

### 3.5. Detection Sensitivity and Selectivity

**Figure 5:**
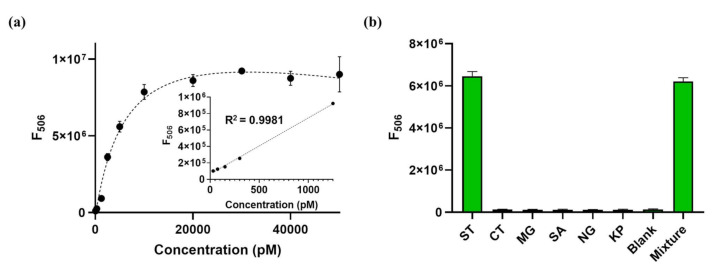
Detection performance of the new detection system. (**a**) Detection sensitivity of the new detection system. Inset shows the linear range between fluorescence intensity at 506 nm (F_506_) and target nucleic acid concentration (0–1250 pM). (**b**) Detection selectivity of the new detection system. The target nucleic acid is from *Salmonella* Typhi (ST), whereas the non-target nucleic acids are from *Chlamydia trachomatis* (CT), *Mycoplasma genitalium* (MG), *Staphylococcus aureus* (SA), *Neisseria gonorrhoeae* (NG), and *Klebsiella pneumonia* (KP). The final concentration of both target and non-target nucleic acids is 10 nM. All experiments were performed in triplicate.

The sensitivity of the new detection system was determined by measuring the fluorescence emission intensity at 506 nm (F_506_), which is the emission maximum of DFHBI, in the presence of varying concentrations of the target nucleic acids. As shown in Figure 5a, the fluorescence signal increased as the target nucleic acid concentration was increased to 10 nM, at which concentration the signal was saturated. A linear relationship was observed in the range of 50–1250 pM (F_506_ = 682.4 × C + 64414) with a correlation coefficient (R^2^) of 0.9981. The limit of detection (LOD) was estimated to be 37.6 pM based on the definition of LOD = 3σ/S, where σ and S are the standard deviation of the blank and the slope of the linear relationship, respectively. This is comparable to those from previous strategies for the detection of target nucleic acids [5,41,42,43,44,45,46].

Non-target DNAs, including artificially synthesized nucleic acids designed from whole genomes of *Mycoplasma genitalium* (MG), *Staphylococcus aureus* (SA), *Neisseria gonorrhoeae* (NG), *Klebsiella pneumonia* (KP), and *Chlamydia trachomatis* (CT), were employed to evaluate the detection selectivity of the proposed method. As shown in Figure 5b, fluorescence intensity substantially increased only in the presence of the target DNA from ST, whereas other non-target DNAs generated a negligible fluorescence signal. These results indicate that the sensor is highly selective for the target nucleic acids.

### 3.6. Practical Applicability

**Figure 6:**
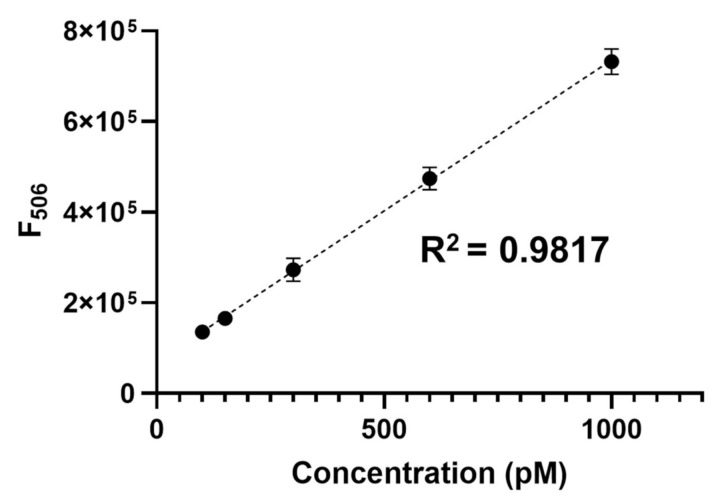
Linear relationship between the fluorescence intensity (F_506_) and concentration of target nucleic acids spiked in the diluted human serum (1%). All experiments were performed in triplicate.

Finally, the practical applicability of the new detection system was demonstrated by performing spike-and-recovery experiments in human serum [47]. Specifically, mock clinical samples were prepared by spiking various concentrations of target DNA (ST) into human serum, which was then analyzed using the proposed 3WJ-induced transcription amplification strategy. As shown in Figure 6, the F_506_ increased linearly at target nucleic acid concentrations between 100 and 1000 pM (R^2^ = 0.9817). Importantly, the target nucleic acid concentrations spiked in human serum were accurately determined, as evidenced by a coefficient of variation (CV) < 1% and a recovery rate of 99–101% (Table 2). These results confirm the excellent reproducibility and accuracy of this method, demonstrating the possibility of detecting target nucleic acids in clinical samples.

## 4. Conclusions

We devised an advanced strategy for the detection of target nucleic acids that relies on 3WJ-induced transcription amplification. The S/B ratio was markedly improved by rationally adopting light-up RNA aptamers compared to that obtained with the use of common DNA staining dyes, allowing for reproducible analysis of target nucleic acids with the suppression of false positive signals. In addition, the whole process was executed at 37 °C, and the reaction conditions were optimized for efficient analysis of target nucleic acids. With the proposed system, we quantitatively analyzed target nucleic acids with high selectivity. Furthermore, the practical applicability of the method was proven by determining spiked levels of target nucleic acids in human serum. We expect that this new method can be applied to detect other nucleic acid-based biomarkers by simply replacing the target specific regions.

## Figures and Tables

**Table 1:** Oligonucleotide sequences used in this study.

Name	Sequence (5′→3′)
*Salmonella* Typhi (ST) = Target nucleic acids	ACT GGC GTT ATC CCT TTC TCT GGT GCT GGC ATT TTC CAG
3WJ_template	TAA TAC GAC TCA CTA TAG GGC GGG AGA AGG ACG GGT CCA GCG TTC GCG CTG TTG AGT AGA GTG TGA GCT CCC TAA TGA TCC CAT AAT CAA AGG GAT AAC GCC AGT GGG TAA TAC GAC TCA CTA TAG GG-phosphate
3WJ_primer (3P)	CTG GAA AAT GCC AGC ACC AGA GTT GAT
3WJ_primer (4P)	CTG GAA AAT GCC AGC ACC AGA GTT GAT T
3WJ_primer (5P)	CTG GAA AAT GCC AGC ACC AGA GTT GAT TA
3WJ_primer (6P)	CTG GAA AAT GCC AGC ACC AGA GTT GAT TAT
3WJ_primer (7P)	CTG GAA AAT GCC AGC ACC AGA GTT GAT TAT G
*Chlamydia trachomatis* (CT)	CGT GCG GGG TTA TCT TAA AAG GGA TTG CAG CTT GTA GTC
*Mycoplasma genitalium* (MG)	CAA GTA TCT CAA GTA TCT CAA TGC TGT TGA GAA ATA CCT
*Staphylococcus aureus* (SA)	ATG ACA TTC AGA CTA TTA TTG GTT GAT ACA CCT GAA ACA
*Neisseria gonorrhoeae* (NG)	ATC AAC CCT GCC GCC GAT ATA CCT AGC AAG CTC CAC AGA
*Klebsiella pneumoniae* (KP)	GGT CGG CGA ACT CTG CGC CGC CGC CAT TAC CAT GAG CGA

**Table 2:** Determination of target nucleic acid concentrations in human serum.

Added (pM) ^a^	Measured (pM) ^b^	SD ^c^	CV (%) ^d^	Recovery (%) ^e^
200	199.2	0.888	0.446	99.6
500	503.1	1.87	0.372	101

^a^ A calibration curve was first created using standards containing known concentrations of the target nucleic acids spiked in diluted human serum (1%) (Figure 6). Based on the calibration curve, the F_506_ from the unknown sample was used to determine the target nucleic acid concentration in human serum. ^b^ Mean of three measurements. ^c^ SD, Standard deviation of three measurements. ^d^ CV, Coefficient of variation = SD/mean × 100. ^e^ Measured value/added value × 100.
